# Promoter mutations and cellular distribution of telomerase in non-clear cell and clear cell hepatocellular carcinoma

**DOI:** 10.18632/oncotarget.15458

**Published:** 2017-02-17

**Authors:** Wan Huang, Weiping Zhou, Can Li, Yuan Yang, Yu-Kui Shang, Changsheng Chen, Jing Zhang, Rui Yao, Pei Wang, Wen Wen, Han-Qiang Liu, Ling Wang, Xia Li, Huijie Bian, Zhi-Nan Chen

**Affiliations:** ^1^ National Translational Science Center for Molecular Medicine, Department of Cell Biology, Fourth Military Medical University, Xi'an, China; ^2^ The Third Department of Hepatic Surgery, Eastern Hepatobiliary Surgery Hospital, Second Military Medical University, Shanghai, China; ^3^ Department of Health Statistics, Fourth Military Medical University, Xi'an, China; ^4^ Department of Pathology, Xijing Hospital, Fourth Military Medical University, Xi'an, China; ^5^ Department of Nutrition and Food Hygiene, Fourth Military Medical University, Xi'an, China

**Keywords:** telomerase, subcellular localization, mutation, non-clear cell hepatocellular carcinoma, clear cell hepatocellular carcinoma

## Abstract

Reactivation of telomerase is a critical step in the development of hepatocellular carcinoma (HCC). Here we identified the frequency of mutations in telomerase reverse transcriptase (TERT) promoter was 34% in non-clear cell HCC (NCCHCC, *n* = 259) and 26.3% in clear cell HCC (CCHCC, *n* = 57). The mutations were independently associated with poor recurrence-free survival of HCCs. Interestingly immunohistochemical analysis demonstrated a higher positive rate of TERT cytoplasmic localization (95%) than nuclear localization (64%) in HCCs. In NCCHCCs, the mutations correlated with higher TERT nuclear expression and increased telomere-dependent telomerase activity. Higher cytoplasmic expression was found in adjacent tissues compared to tumor tissues, and was associated with tumor well-differentiation and lower level of α-fetoprotein. NCCHCCs with low nuclear as well as high cytoplasmic expression correlated with better prognosis. In CCHCCs, elevated TERT cytoplasmic expression was observed in CCHCCs harboring mutations. Higher TERT cytoplasmic expression was found in tumor tissues compared to adjacent tissues, and was associated with multiple numbers of tumors and poor prognosis of CCHCCs. In conclusion, mutations in TERT promoter disclose the significance of both nuclear and cytoplasmic TERT in HCC. Cytoplasmic TERT should also be considered when determining prognosis and treatment of HCCs.

## INTRODUCTION

Human telomerase reverse transcriptase (TERT), a catalytic subunit of telomerase, plays a critical role in the pathology of aging and cancer by maintaining genome integrity and controlling cell proliferation [1]. Telomerase is reactivated in almost 90% of cancers, which drives cell immortality and carcinogenicity [2]. It is reported that in the precancerous liver cirrhosis, telomere shortening concomitant with a weak positive TERT signal in the nucleus is observed. In late hepatocirrhosis, increased telomerase activity might be a turning point in HCC development [3]. Several studies identify recurrent somatic mutations in TERT promoter in various cancers, including liver cancer [4–7]. The two mutations -124 and -146 base pairs (bp) from the ATG translation start site in TERT promoter, are proposed to augment transcription by generating binding motifs for E-twenty six/ternary complex factors (Ets/TCF) [8, 9]. In addition to canonical telomeric-DNA synthesis activity, telomere-independent functions of telomerase can be performed within the nucleus as well as in other cellular compartments [10]. Cytoplasmic TERT is once dismissed as non-specific staining and non-functional protein. Recent studies confirm the non-nuclear locations of TERT, such as the cytoplasm and mitochondria [11]. However, the expression of cytoplasmic TERT and its impact on clinical progression have not been evaluated in the tumor tissues of HCC.

According to histological features and origin, primary HCC includes particular histological types such as clear cell HCC (CCHCC). CCHCC is previously regarded as an uncommon variant of HCC, but the incidence is high, up to 13.3% and 9.5% of cases in reports from Mainland and Taiwan of China, respectively [12, 13]. Microscopically, CCHCC is characterized by cytoplasmic accumulation of glycogen and/or fat droplets that dissolve during hematoxylin-eosin (H&E) staining, which leaves a clear cytoplasm [14]. The cytoplasmic accumulation of glycogen or lipid is related to the changes in metabolic pathways or metabolic defects within the cells [15]. Although the tumor cells of CCHCC are generally moderate or well differentiation, the incidence of tumor metastasis and recurrence after resection is similar to that of non-clear cell HCC (NCCHCC) [16, 17].

The present study analyzed the promoter mutations and cellular distribution of telomerase in NCCHCC and CCHCC, and disclosed the correlation of TERT subcellular expression with clinical characteristics, which further our understanding of telomerase activity in the progression of HCC.

## RESULTS

### TERT promoter mutations in different subtypes of HCC and the prognostic value of mutations in predicting recurrence of HCC

**Figure 1:**
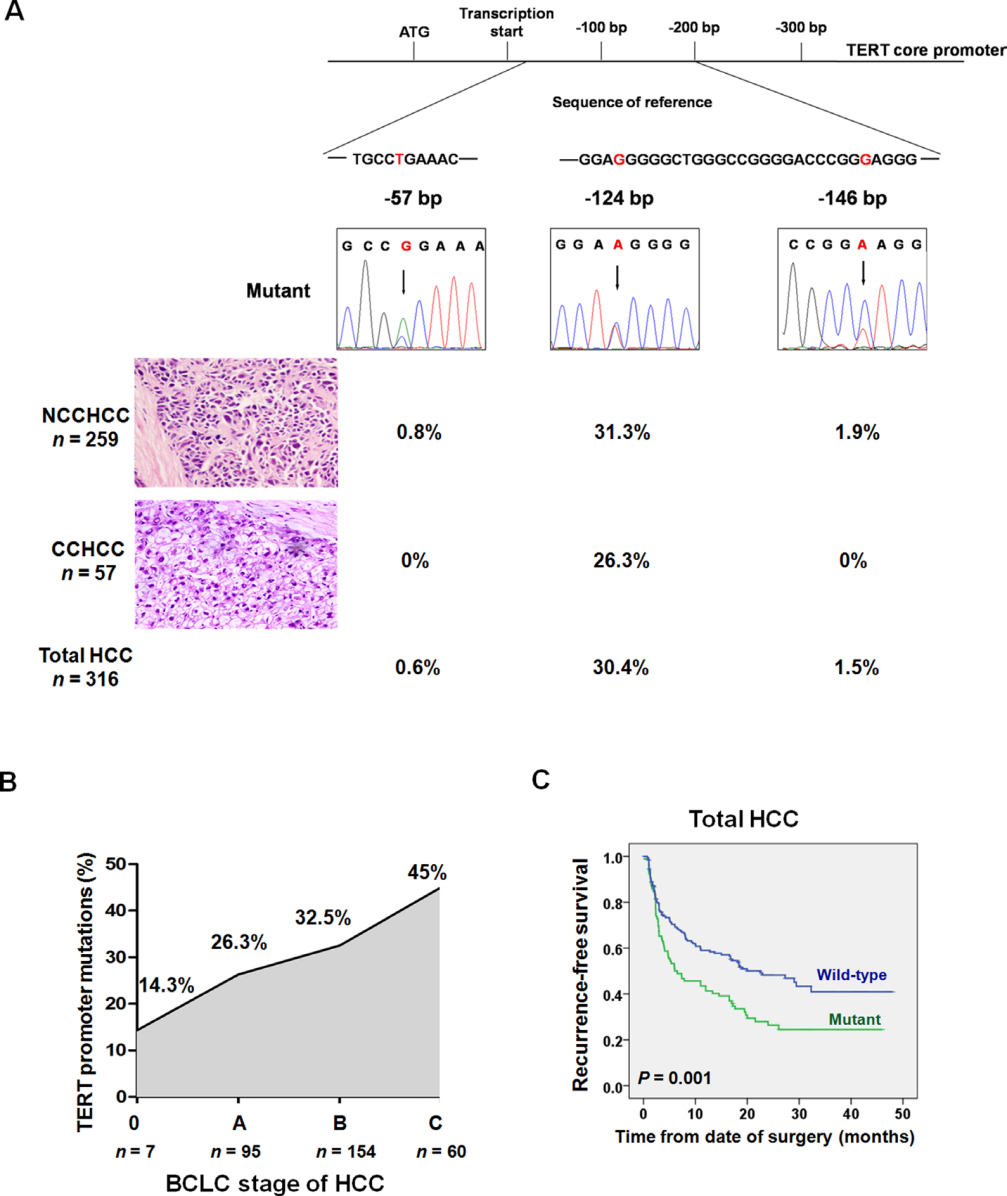
TERT promoter mutations in different subtypes of HCC and the prognostic value of mutations in predicting recurrence after resection (**A**) Frequency of TERT promoter mutations in different subtypes of HCC. (**B**) TERT promoter mutations in the process of HCC progression according to BCLC staging. The frequency was stepwise increased during HCC progression (*P* = 0.038, χ^2^-test). (**C**) Kaplan-Meier plots of disease-free survival curves for patients with primary HCC stratified by TERT promoter mutation status (*n* = 275). Survival analysis was performed using the log-rank test. Abbreviations: BCLC, Barcelona Clinic Liver Cancer; CCHCC, clear cell hepatocellular carcinoma; NCCHCC, non-clear cell hepatocellular carcinoma.

We examined TERT promoter mutations in 316 cases of primary HCC that consisted of NCCHCC (*n* = 259) and CCHCC (*n* = 57). The patient demographics and clinicopathologic features of the cohorts were summarized in Supplementary Table 1. The reported most frequent mutation of TERT promoter, -124 bp, was found in 81 of 259 (31.3%) NCCHCCs and 15 of 57 (26.3%) CCHCCs. (Figure 1A). TERT promoter -146 bp mutation was found exclusively in five of 259 (1.9%) NCCHCCs. Another -57 bp mutation, which was originally discovered as a causal mutation in a melanoma family [9], was detected in two of 259 (0.8%) NCCHCCs. The overall frequency of -124 bp, -146 bp and -57 bp was 30.4%, 1.5% and 0.6% in total HCCs, respectively. Further analysis indicated that TERT promoter mutations were more frequent in men (*P* = 0.026, χ^2^-test) and in patients with older age (*P* = 0.012, unpaired student's *t* test), poor tumor differentiation (*P* = 0.028, χ^2^-test) and advanced Barcelona Clinic Liver Cancer (BCLC) stage (*P* = 0.038, χ^2^-test) (Supplementary Table 2). The multivariate analysis confirmed that older age (*P* = 0.006), advanced BCLC stage (*P* = 0.032), and patients without cirrhosis history (*P* = 0.027) were independent factors associated with TERT promoter mutations (Supplementary Table 3).

Next we analyzed the frequency of TERT promoter mutations in different BCLC stages of patients with HCC. The mutations were identified in 14.3% in stage 0, 26.3 % in stage A, 32.5% in stage B, and 45% in stage C (Figure 1B). The Kaplan-Meier survival analysis indicated that TERT promoter mutations were associated with reduced recurrence-free survival of patients with HCC (*P* = 0.001, Log-rank test) (Figure 1C), which was further confirmed by multivariate analysis after adjustment for classical clinicopathologic risk factors. The hazard ratio (HR) was 1.72 (95% CI: 1.23 to 2.39, *P* = 0.001, Supplementary Table 4).

### Cytoplasmic and nuclear expression of TERT in HCC

**Figure 2:**
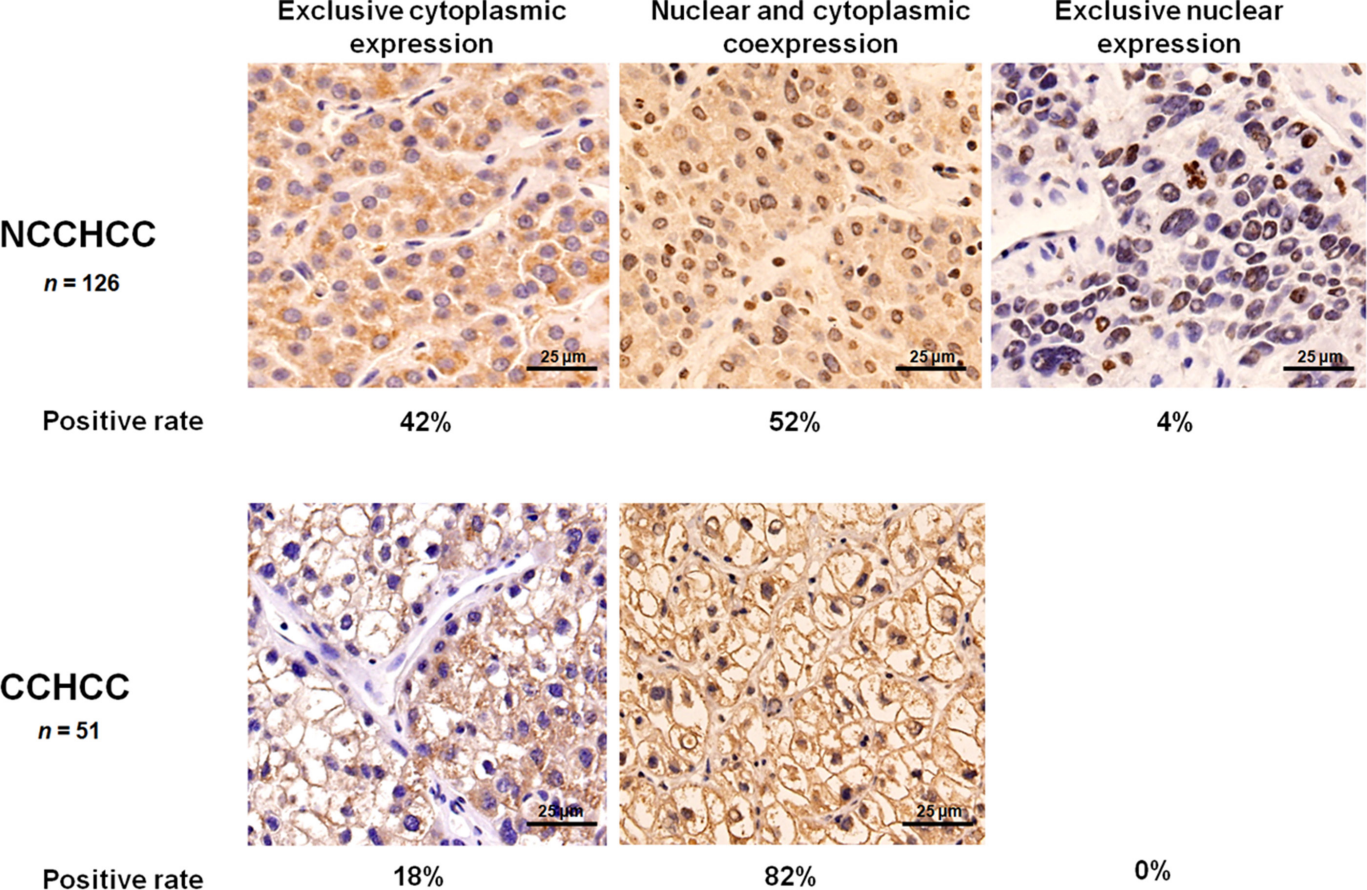
Cytoplasmic and nuclear expression of TERT in HCC Representative images of immunohistochemical staining in NCCHCC and CCHCC, and the positive rate of TERT localized in both cytoplasm and nucleus, exclusively in the cytoplasm, and exclusively in the nucleus.

We detected the subcellular localization of TERT in 126 cases of NCCHCC and 51 cases of CCHCC by immunohistochemistry. As shown in Figure 2, 52% cases of NCCHCC showed both cytoplasmic and nuclear localization of TERT, 42% cases were exclusively cytoplasmic positive, 4% cases were exclusively nuclear positive, and 2% cases were both cytoplasmic and nuclear negative expression. In CCHCC, 82% cases showed both cytoplasmic and nuclear localization of TERT and 18% cases were exclusively cytoplasmic positive. No exclusive nuclear expression was observed. The results demonstrated a high positive rate of TERT cytoplasmic localization (95%) in addition to nuclear localization (64%) in the tumor tissues of HCC. In order to verify the localization and the expression level of TERT subcellular expression which were evaluated by immunohistochemistry, we further used immunofluorescence and western blot to detect TERT expression in cytoplasm and in nucleus, respectively. Representative images of six cases demonstrating different expression level of cytoplasmic and nuclear TERT were shown in Supplementary Figure 1A and 1B, and the results were confirmed by western blot analysis (Supplementary Figure 1C and 1D).

### TERT promoter mutations correlated with telomere-dependent activity in NCCHCC and elevated TERT cytoplasmic expression in CCHCC

**Figure 3:**
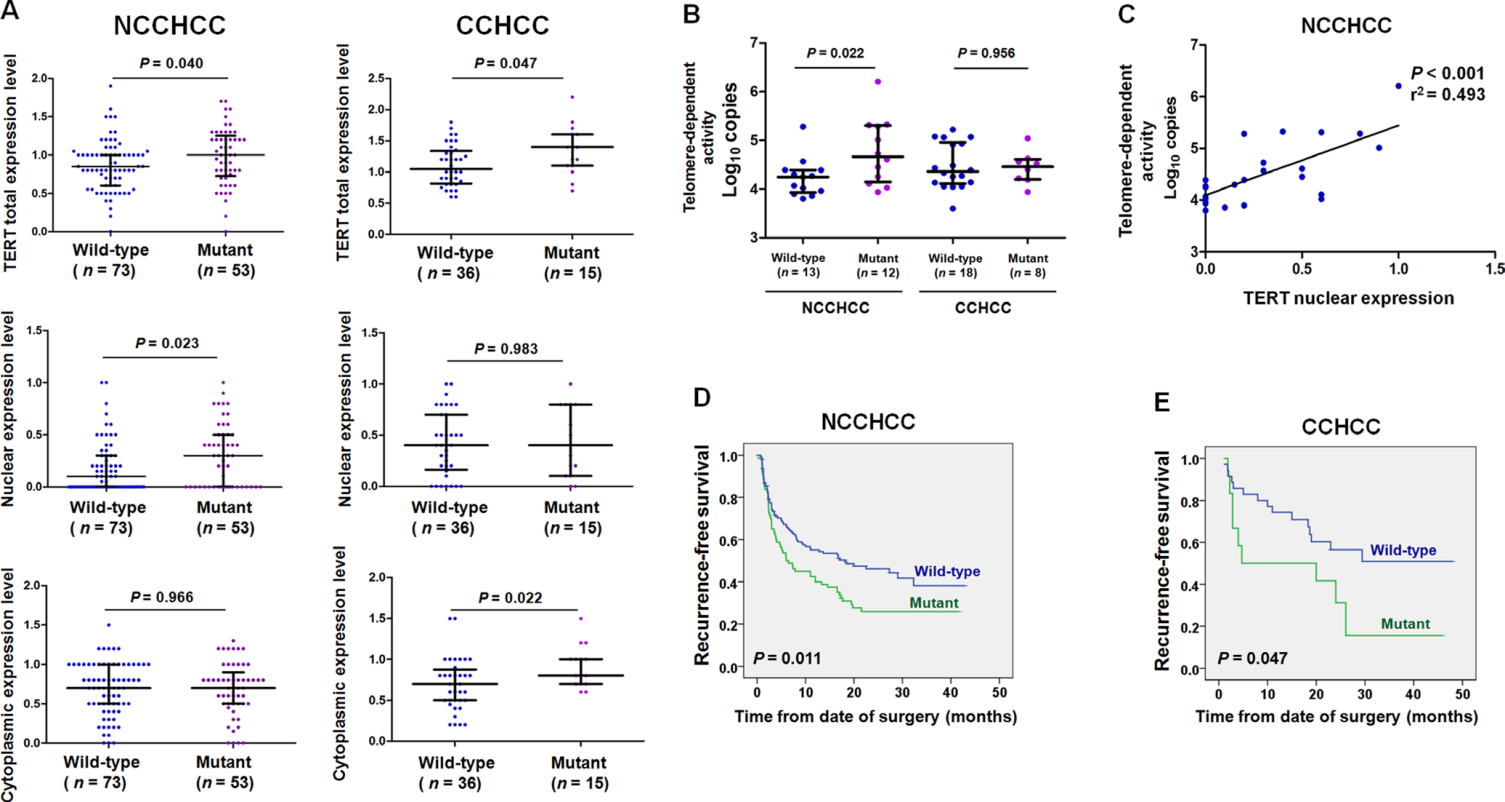
TERT promoter mutations correlated with telomere-dependent activity in NCCHCC and elevated TERT cytoplasmic expression in CCHCC (**A**) Effects of TERT promoter mutations on TERT subcellular expression level in NCCHCCs and CCHCCs. TERT expression in cytoplasm and nucleus were evaluated by immunohistochemical score. Results were reported in median with interquartile range and compared using the non-parametric Mann-Whitney test. (**B**) Effects of TERT promoter mutations on telomere-dependent activity in NCCHCCs and CCHCCs. The results were reported in median with interquartile range and were compared using the non-parametric Mann-Whitney test. (**C**) Telomerase activity correlated with TERT nuclear expression in NCCHCCs. *P* value was derived from Pearson correlation analysis. (**D** and **E**) Kaplan-Meier plots of disease-free survival curves for patients with NCCHCC (*n* = 228), and CCHCC (*n* = 47) stratified by TERT promoter mutation status. Survival analysis was performed using the log-rank test. Abbreviations: BCLC, Barcelona Clinic Liver Cancer.

We then analyzed the effect of mutations on TERT expression. As shown in Figure 3A, TERT promoter mutations were associated with an increased TERT total protein expression level (nuclear and cytoplasmic expression) in both NCCHCCs and CCHCCs (*P* = 0.040 and *P* = 0.047, respectively, Mann-Whitney test). Interestingly, TERT nuclear expression level was significantly higher in NCCHCCs harboring mutant than that in wild-type cases (*P* = 0.023, Mann-Whitney test). In contrast, TERT cytoplasmic expression level was significantly elevated in CCHCCs harboring mutations compared to that in wild-type (*P* = 0.022, Mann-Whitney test).

In order to verify the hypothesis that TERT promoter mutation was correlated with telomere-dependent activity in NCCHCCs, we quantified the telomerase activities in tumor tissues by TRAP (telomeric repeat amplification protocol) assay. The results in Figure 3B indicated that the telomere-dependent activity in NCCHCCs harboring mutations was significantly raised compared with that in wild-type cases (*P* = 0.022, Mann-Whitney test), whereas no significant difference of telomere-dependent activity was found between TERT wild-type and mutant in CCHCCs (*P* = 0.956, Mann-Whitney test). Pearson correlation analysis in Figure 3C indicated that telomerase activity correlated with TERT nuclear expression in NCCHCC patients (*P* < 0.001, r^2^ = 0.493). The results revealed that increased nuclear expression of TERT in NCCHCCs harboring TERT promoter mutations was accompanied by enhanced telomerase activity in nucleus. The Kaplan-Meier survival analysis indicated that TERT promoter mutations were associated with reduced recurrence-free survival of patients with both NCCHCCs (*P* = 0.011, Log-rank test, Figure 3D) and CCHCCs (*P* = 0.047, Log-rank test, Figure 3E).

### TERT subcellular expression in paired adjacent and tumor tissues of NCCHCC and CCHCC

**Figure 4:**
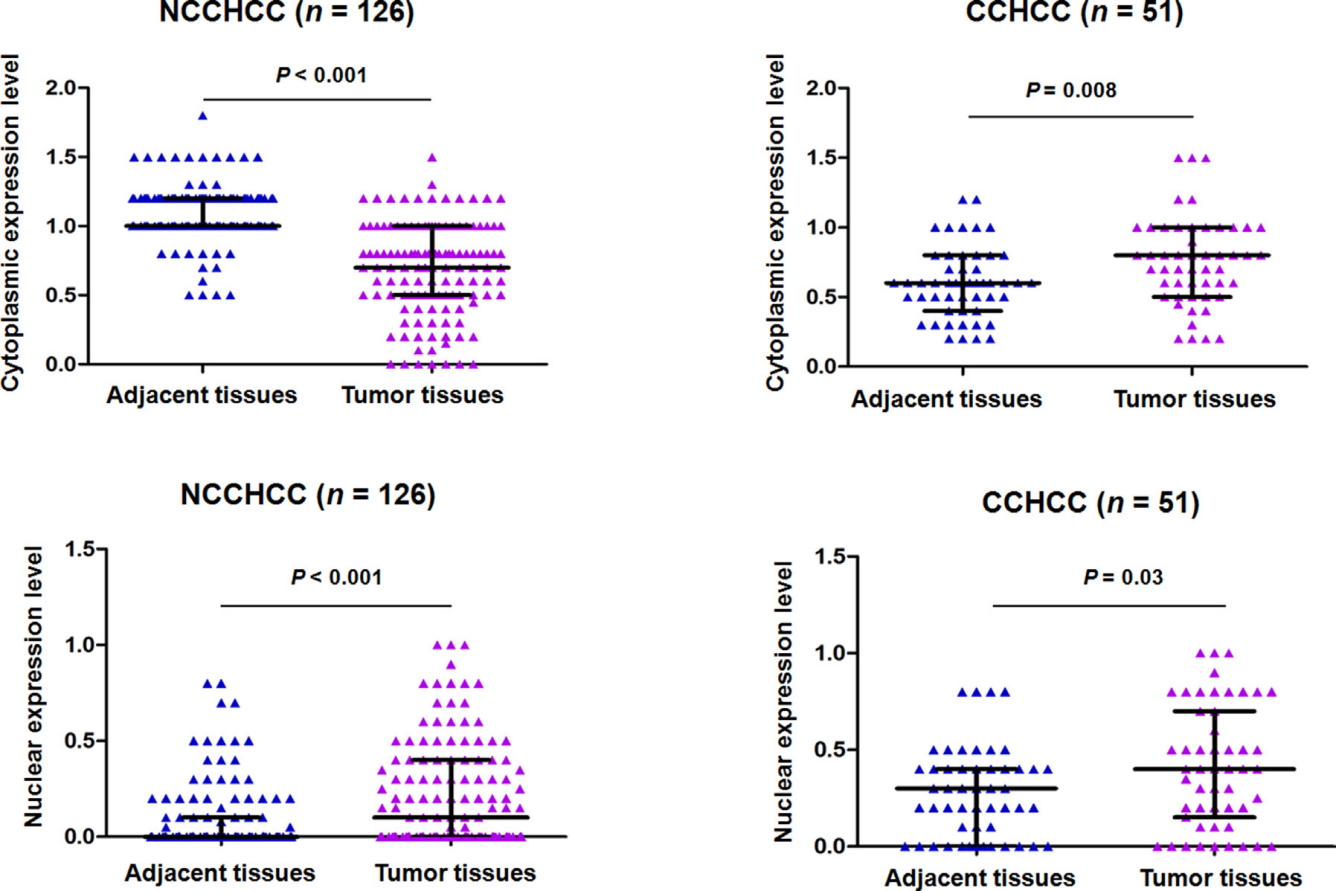
TERT subcellular expression in paired adjacent and tumor tissues of NCCHCC and CCHCC TERT expression in cytoplasm and nucleus was evaluated by immunohistochemical score for each sample. Statistical results were reported in median with interquartile range and were compared using the non-parametric Mann-Whitney test.

We analyzed the TERT subcellular expression in paired adjacent and tumor tissues of NCCHCC and CCHCC by immunohistochemistry. The analysis of immunohistochemical scoring in Figure 4 showed that in NCCHCCs, the TERT cytoplasmic expression level in tumor tissues was much lower than that in adjacent tissues (*P* < 0.001, Mann-Whitney test), whereas TERT nuclear expression level was significantly increased in tumor tissues compared with that in adjacent tissues (*P* < 0.001, Mann-Whitney test). In CCHCC, both TERT cytoplasmic and nuclear expression levels in tumor tissues were significantly higher than that in corresponding adjacent tissues (*P* = 0.008 and *P* = 0.03, respectively, Mann-Whitney test).

### Association of TERT subcellular expressions with clinical characteristics and recurrence-free survival in NCCHCC and CCHCC

**Figure 5:**
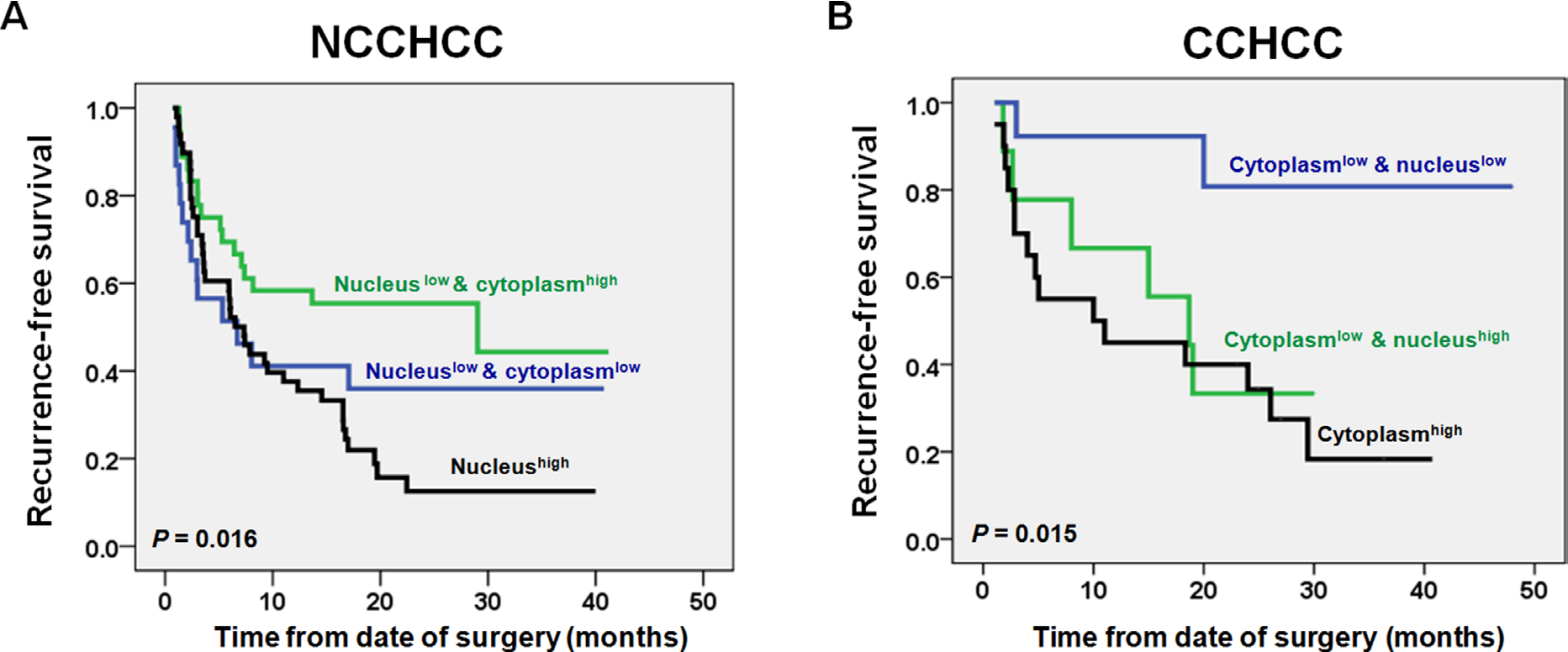
Impacts of TERT nuclear and cytoplasmic expressions on recurremce-free survival of patients with NCCHCC and CCHCC Kaplan-Meier plots of recurrence-free survival curves for NCCHCC (*n* = 109) and CCHCC (*n* = 42) (**A** and **B**) patients stratified by the level of TERT cytoplasmic and nuclear expression. Survival analysis was performed using the log-rank test.

We compared the TERT nuclear and cytoplasmic expressions between different subgroups stratified by clinical characteristics in NCCHCCs and CCHCCs, respectively (Table 1). In NCCHCCs, higher expression level of nuclear TERT was significantly associated with multiple number of tumors (*P* = 0.002, Mann-Whitney test). Interestingly, higher expression level of cytoplasmic TERT was significantly associated with lower level of α-fetoprotein and tumor well-differentiation (*P* = 0.028 and *P* = 0.021, respectively, Mann-Whitney test, Table 1). The Kaplan-Meier survival analysis further demonstrated that NCCHCCs with low nuclear as well as high cytoplasmic expression tended to have a better recurrence-free survival, whereas NCCHCCs with high nuclear expression had the worst prognosis (*P* = 0.016, Log-rank test, Figure 5A). The results above indicated that cytoplasmic TERT correlated with early development of NCCHCCs, whereas nuclear TERT correlated with aggressive behavior and poor survival of NCCHCCs.

**Table 1:** Comparison of TERT nuclear and cytoplasmic expressions between different subgroups of HCCs stratified by host characteristics

Variables	NCCHCC (*n* = 126)				CCHCC (*n* = 51)			
	Nuclear expression	*P*	Cytoplasmic expression	*P*	Nuclear expression	*P*	Cytoplasmic expression	*P*
Gender		0.810		0.983		0.137		0.335
Male	0.1 (0–1.0)		(0–1.5)		0.375 (0–1.0)		0.8 (0.2–1.5)	
Female	0.2 (0–0.5)		0.65 (0–1.6)		0.5 (0–1.0)		0.6 (0.2–1.5)	
Age (years)		0.788		0.645		0.062		0.089
< 55	0.10 (0–1.0)		0.7 (0–1.6)		0.25 (0–0.8)		0.8 (0.2–1.5)	
≥ 55	0.025 (0–1.0)		0.8 (0.1–1.3)		0.4 (0–1.0)		0.7 (0.2–1.2)	
Cirrhosis		0.535		0.052		0.331		0.581
Yes	0.05 (0–0.7)		0.8 (0–1.5)		0.4 (0–1.0)		0.6 (0.2–1.5)	
No	0.15 (0–1.0)		0.7 (0–1.6)		0.2 (0–0.5)		0.8 (0.2–1.5)	
α-Fetoprotein (μg/L)		0.440		0.028*		0.329		0.984
≤ 200	0.05 (0–0.8)		0.8 (0–1.5)		0.4 (0–1.0)		0.75 (0.2–1.5)	
> 200	0.1 (0–1.0)		0.7 (0–1.6)		0.2 (0–1.0)		0.8 (0.2–1.5)	
Tumor differentiation		0.669		0.021*		0.523		0.626
Well and Moderate	0.1 (0–0.8)		0.8 (0–1.6)		0.45 (0–1.0)		0.8 (0.2–1.5)	
Poor	0.1 (0–1.0)		0.6 (0–1.5)		0.4 (0–1.0)		0.7 (0.2–1.5)	
BCLC stage		0.111		0.808		0.031*		0.814
0 and A	0 (0–1.0)		0.7 (0–1.6)		0.55 (0–1.0)		0.7 (0.2–1.5)	
B and C	0.15 (0–1.0)		0.7 (0–1.5)		0.3 (0–0.8)		0.8 (0.2–1.5)	
Tumor size (cm)		0.259		0.355		0.039*		0.242
< 5	0 (0–1.0)		0.7 (0–1.6)		0.5 (0–1.0)		0.75 (0.2–1.5)	
≥ 5	0.15 (0–0.9)		0.8 (0–1.5)		0.3 (0–0.8)		0.8 (0.2–1.5)	
Portal vein thrombosis		0.735		0.642		0.348		0.978
Yes	0.1 (0–1.0)		0.7 (0–1.6)		0.15 (0–0.8)		0.7 (0.3–1.5)	
No	0.05 (0–0.8)		0.65 (0–1.3)		0.4 (0–1.0)		0.8 (0.2–1.5)	
Number of tumors		0.002*		0.898		0.848		0.031*
Single	0 (0–1.0)		0.7 (0–1.6)		0.4 (0–1.0)		0.7 (0.2–1.2)	
Multiple	0.3 (0–1.0)		0.7 (0–1.5)		0.4 (0–0.8)		0.9 (0.5–1.5)	
Family history		0.823		0.145		0.364		0.627
Yes	0.025 (0–1.0)		1 (0–1.5)		0.3 (0–1.0)		0.8 (0.2–1.5)	
No	0.1 (0–1.0)		0.7 (0–1.6)		0.45 (0–1.0)		0.75 (0.2–1.5)	

TERT nuclear and cytoplasmic expressions were reported in median with interquartile range of immunohistochemical score. *P* values were derived from the non-parametric Mann-Whitney test. * indicates statistical significant (*P* < 0.05). Abbreviations: BCLC, Barcelona Clinic Liver Cancer.

In CCHCCs, the higher expression level of cytoplasmic TERT was associated with multiple numbers of tumors (*P* = 0.031, Mann-Whitney test, Table 1). Whereas higher expression level of nuclear TERT was associated with early BCLC stage and smaller tumor size (*P* = 0.031 and *P* = 0.039, Mann-Whitney test). The Kaplan-Meier survival analysis showed that CCHCCs with low nuclear as well as low cytoplasmic expression had a better prognosis, whereas CCHCCs with high cytoplasmic expression had the worst recurrence-free survival (*P* = 0.015, Log-rank test, Figure 5B). The results demonstrated that cytoplasmic TERT was correlated with aggressive feature and poor prognosis of CCHCCs.

## DISCUSSION

The discovery of the somatic mutations activating TERT promoter in several tumor types adds a new perspective to reevaluate the anti-telomerase strategies [4–9]. Previous studies reveal that as an early somatic genetic alteration in hepatocarcinogenesis, TERT promoter mutations are identified in dysplastic nodules and early HCC [18, 19]. In our cohort that 85% of patients had a history of HBV infection, we demonstrated that the frequency of mutations was also stepwise increased during advanced HCC progression (with the highest frequency of 45% in patients with stage C). The survival analysis further validated that the mutations could be used as a prognostic marker for HCC, which was consistent with the study by Kawai-Kitahata F *et al*. [20].

By recruiting ETS transcription factors to newly generated motifs, TERT promoter mutations are proposed to augment transcription of TERT [21]. In accordance with this, we found that the mutations correlated with higher levels of TERT protein expression. Interestingly, we also observed that elevated TERT expression exhibited subcellular distribution characteristics in different histological types of HCC. Enhanced nuclear expression was found in NCCHCCs and augmented cytoplasmic expression was found in CCHCCs. The shuttle of TERT between nucleus and cytoplasm in tumor cells could be regulated by certain mechanisms such as phosphorylation, depending on cell cycle or cellular immortalized transformation [22–24].

In addition to canonical telomere-dependent activity of TERT in the nucleus, telomere-independent activities of TERT are involved in many biological processes including regulation of gene expression and mitochondrial functionality [10]. Although several studies reveal the impact of mitochondrial translocation of telomerase on drug resistance in HCC cell lines [25–26], the cellular distribution and telomere-independent activities of TERT has not been investigated in tumor tissues of HCC patients. In this study, we found TERT positively expressed in the nucleus in 64% of HCCs, and the positive rate of telomere-dependent activity was 60%, which was a little bit lower than that in previous reports that telomere-dependent activity was found in 61.5%–95.8% of HCC tissues [27–29]. In contrast, we observed a high percentage (95%) of TERT cytoplasmic localization. We considered this could be explained by different cohort. In Chinese population, the most common risk factor in the development of HCC is chronic HBV infection, which is an important factor that contributes to oxidative stress [30, 31]. Moreover, cycoplasmic TERT has been shown to improve mitochondrial function and contribute to higher resistance of cancer cells against DNA damage induced by oxidative stress [32–34]. Therefore, the high positive rate of cytoplasmic TERT in HCC was probably a response to continuous oxidative stress caused by HBV infection.

Intriguingly, the roles of cytoplasmic TERT might be different between NCCHCC and CCHCC. In NCCHCC, we found that TERT cytoplasmic expression was significantly higher in adjacent tissue and was associated with better tumor differentiation. We considered that in precancerous stage, the high expression of TERT in cytoplasm increases resistance against HBV induced oxidative stress. In the process of HCC development, cytoplasmic TERT was decreased either by inhibiting nuclear export or by enhancing the nuclear import of TERT, whereas increased nuclear localization facilitated cell proliferation. We provide evidences in human tissue samples that cytoplasm-to-nucleus translocation was a critical process in the malignant transformation of NCCHCC, which was agreement with a previous study conducted in chemical induced rat HCC [35]. In CCHCC, our data demonstrated that TERT cytoplasmic expression elevated in the malignant alteration, correlated with multiple numbers of tumors and tumor recurrence. Characterized by abundant accumulation of lipid and glycogen in the cytoplasm, CCHCC is related to abnormal cell metabolism. The study on TERT knockout mice reveals that telomere dysfunction is associated with decreased gluconeogenesis [36]. Furthermore, another study demonstrates that extra-nuclear TERT is associated with glucose transporters and regulated glucose transport [37]. Therefore, we hypothesize that the elevated cytoplasmic TERT in CCHCC is responding to abnormal glucose or lipid metabolism, thereby inhibiting cell apoptosis and promoting cancer cell survival.

In summary, the mutations in TERT promoter correlate with telomere-dependent activities in NCCHCCs and elevated cytoplasmic expression in CCHCCs. The high positive rate of TERT cytoplasmic expression provides novel perspective for anti-telomerase treatment of HCCs. Inhibiting cytoplasm-to-nucleus translocation could be a more effective strategy for patients with NCCHCC.

## MATERIALS AND METHODS

### Patients and analysis of TERT promoter mutations

From January 2008 to November 2011, a total of 316 tissues of primary HCC resected from patients (Eastern Hepatobiliary Surgery Hospital, Shanghai) were used in this study. All cases were confirmed with primary HCC and had no previous history of other cancers or cancer-related treatments prior to surgery. All resected tumors underwent pathological assessment, and tissues with necrosis area ≥ 20% or area of tumor tissue ≤ 70% were excluded. All patients received regular follow-up examinations. The censored data were those for patients without relapse or death at the last follow-up. The anonymity of each patient was maintained, and all specimens were blindly analyzed. The study was approved by the Institutional Review Boards of Fourth Military Medical University, and the signed informed consent was obtained from each participant. The methods were carried out in accordance with the approved guidelines.

Genomic DNA samples were isolated from frozen tissues using the TIANamp Genomic DNA Kit (TIANGEN, Beijing, China). A 488 bp region of TERT promoter region encompassing the mutational hotspots was amplified with forward primer 5′-AGCACCTCGCGGTAGTGG-3′ and reverse primer 5′-GGCCGATTCGACCTCTCT-3′. The PCR was performed at an initial temperature of 94°C for 1 min, followed by 40 cycles at 94°C for 30 s, 62°C for 30 s, 72°C for 50 s, and finally, 72°C for 5 min. After purification, the TERT promoter was sequenced using the Sanger technique.

### Immunohistochemistry and immunofluorescence

Paraffin-embedded tissue samples were cut into 4 μm sections. Immunohistochemical and immunofluorescence staining for TERT was performed with a mouse monoclonal antibody (diluted 1:100, Abcam, Cambridge, USA). The results of immunofluorescence staining were observed by A1 laser scanning confocal microscope (Nikon Instruments, New York, USA). The results of immunohistochemical staining were independently evaluated by two experienced pathologists blind to the study design. The immunohistochemical score was defined based on both the intensity of the staining and the percentage of stained cells. A proportion score was represented by the percentage of positively stained tumor cells (0%–100%). An intensity score, which represented the average intensity of the positive tumor cells, was estimated based on the intensity compared with the positive control (a typical tumor tissue of HCC with TERT positive expression). Generally, intensity score < 1 represented an intensity lower than the positive control, intensity score = 1 represented an intensity equal to the positive control, and an intensity score > 1 represented an intensity higher than the positive control. The proportion and intensity scores were then multiplied to obtain a total score. For example, if 100% of cells were positively stained in a slide, among which 40% of the positive cells had an intensity of 0.8 and 60% of the positive cells had an intensity of 1.2, then the total score was defined as (0.4 × 0.8) + ( 0.6 × 1.2) = 1.04.

### Nuclear and cytoplasmic protein extraction and western blot analysis

Nuclear and cytoplasmic fractionations of tumor tissues of HCC were extracted using the NE-PER Nuclear and Cytoplasmic Extraction Reagents kit (Thermo Fisher Scientific, Rockford, USA) according to the manufacturer's protocol. Protein concentration was quantified using Pierce BCA Protein Assay. Ten microgram of each cytoplasmic and nuclear extract sample was analyzed for western blot with TERT antibody (diluted 1:500, Thermo Fisher Scientific, Rockford, USA), laminB1 antibody (diluted 1:1000, Proteintech, Hubei, China) and β-actin antibody (diluted 1:1000, HuaAn Biotechnology, Zhe Jiang, China). β-actin was used as cytoplasmic loading control for cytoplasmic extracts and laminB was used as nuclear loading control for nuclear extracts.

### Telomerase enzymatic activity analysis

The frozen samples were lysed to release crude cell protein on ice in TRAPeze^®^ 1 × CHAPS lysis buffer (Millipore, Boston, USA). After quantitation, 1 μg of protein lysate was used for each telomerase activity assay using TRAPeze^®^ RT Telomerase Detection Kit (Millipore, Boston, USA). Briefly, the PCR was conducted in a volume of 12.5 μl including 2.5 μl of 5 × TRAPeze^®^ RT Reaction Mix, 8.8 μl of nuclease-free water, 0.2 μl of Taq polymerase, and 1 μl of the protein sample. A dilution series of TSR8 control template was to serve as a standard curve. Reactions were performed in triplicate. The thermal cycling conditions comprised one cycle for 30 min at 30°C, 2 min at 95°C, followed by 45 cycles of 15 s at 94°C, 1 min at 59°C, and 10 s at 45°C. According to the protocol, the positive telomerase activity should be above background at a dilution of 0.04 amols (Log copy number = 4.38) of TSR8 control template.

### Statistical analysis

All statistical analyses were performed using SPSS Statistics version 18.0 software (Chicago, USA). Dichotomous data, along with independence and measures of association, were analyzed using the chi-square test. Continuous/quantitative data were compared using the Mann-Whitney test. Cumulative survival time was calculated using the Kaplan-Meier procedure. The difference in cumulative survival between different groups was analyzed using a log-rank test. Hazard ratios (HR) and 95% confidence intervals (CI) were estimated from a multivariate Cox proportional hazards model. All tests in this study were two-sided and *P* < 0.05 was considered statistically significant.

## SUPPLEMENTARY MATERIALS FIGURES AND TABLES


