# Supplementary material for: Promoter mutations and cellular distribution of telomerase in non-clear cell and clear cell hepatocellular carcinoma
Source: Oncotarget. 2017 Feb 17;8(16):26288–97. doi: 10.18632/oncotarget.15458 (PMC5432257; doi:10.18632/oncotarget.15458)
Supplement: Supplementary file 1 [file oncotarget-08-26288-s001.pdf]

Promoter mutations and cellular distribution of telomerase in non-clear cell and clear cell hepatocellular carcinoma

Supplementary Materials

Supplementary Table 1: Clinical characteristics of the enrolled patients with primary HCC (*n* = 316)

Variables	Number of patients (%)
Age (years)	
< 55	180 (57)
≥ 55	136 (43)
Gender	
Male	268 (85)
Female	48 (15)
HBsAg	
Positive	270 (85)
Negative	46 (15)
Liver cirrhosis	
Yes	231 (73)
No	85 (27)
BCLC stage	
0 and A	102 (32)
B	154 (49)
C	60 (19)
Tumor size (cm)	
< 5	135 (43)
≥ 5	181 (57)
Portal vein thrombosis	
Yes	45 (14)
No	271 (86)
Number of tumors	
Single	255 (81)
Multiple	61 (19)
α-Fetoprotein (μg/L)	
< 200	164 (52)
≥ 200	152 (48)
Family history	
Yes	36 (11)
No	280 (89)

Abbreviations: BCLC, Barcelona Clinic Liver Cancer.

Supplementary Table 2: Characteristics of patients with HCC (n = 316) according to the status of TERT promoter mutations

Variables	TERT wild-type (n = 213)	TERT mutant (n = 103)	P
Gender			
Male (n = 268)	174 (65%)	94 (35%)	0.026*
Female (n = 48)	39 (81%)	9 (19%)	
Age (years), mean ± SD	50.9 ± 11.6	54.2 ± 10.1	0.012*
HBsAg			
Negative (n = 46)	29 (63%)	17 (37%)	0.495
Positive (n = 270)	184 (68%)	86 (32%)	
HBV-DNA (IU/ml)			
≤ 2000 (n = 218)	140 (64%)	78 (36%)	0.432
> 2000 (n = 42)	30 (71%)	12 (29%)	
Cirrhosis			
Yes (n = 224)	156 (70%)	68 (30%)	0.157
No (n = 85)	52 (61%)	33 (39%)	
α-Fetoprotein (μg/L)			
≤ 200 (n = 164)	104 (63%)	60 (37%)	0.116
> 200 (n = 152)	109 (72%)	43 (28%)	
Tumor differentiation			
Well (n = 23)	20 (87%)	3 (13%)	0.028*
Moderate (n = 137)	97 (71%)	40 (29%)	
Poor (n = 156)	96 (62%)	60 (38%)	
BCLC stage			
0 and A (n = 102)	76 (75%)	26 (25%)	0.038*
B (n = 154)	104 (68%)	50 (32%)	
C (n = 60)	33 (55%)	27 (45%)	
Tumor size (cm)			
< 5 (n = 135)	92 (68%)	43 (32%)	0.808
≥ 5 (n = 181)	121 (67%)	60 (33%)	
Portal vein thrombosis			
Yes (n = 45)	26 (58%)	19 (42%)	0.137
No (n = 271)	187 (69%)	84 (31%)	
Number of tumors			
Single (n = 255)	173 (68%)	82 (32%)	0.734
Multiple (n = 61)	40 (66%)	21 (34%)	
Family history			
Yes (n = 36)	23 (64%)	13 (36%)	0.633
No (n = 280)	190 (68%)	90 (32%)	
TERT nuclear expression			
Positive (n = 113)	67 (59%)	46 (41%)	0.405
Negative (n = 64)	42 (66%)	22 (34%)	
TERT cytoplasmic expression			
Positive (n = 169)	105 (62%)	64 (38%)	0.486#
Negative (n = 8)	4 (50%)	4 (50%)	

Data were expressed as number (%) and were analyzed using the $\chi 2$-test except that the P value of Age (years) was calculated using an unpaired student's t test. One hundred and seventy seven cases were used for TERT nuclear and cytoplasmic localization analysis. * indicates statistical significant ($P < 0.05$). # indicates the P value was calculated from Fisher's exact test. *Abbreviations*: BCLC, Barcelona Clinic Liver Cancer.

Supplementary Table 3: Multivariate analysis of clinical pathological factors associated with TERT promoter mutations in patients with HCC (n = 316)

Variables	Odds ratio	95.0% CI	P
Age (years)			
< 55	1.00		
≥ 55	2.24	1.26 to 4.00	0.006*
Gender			
Female	1.00		
Male	2.00	0.85 to 4.71	0.112
α-Fetoprotein (μg/L)			
≤ 200	1.00		
> 200	0.69	0.38 to 1.25	0.222
Tumor differentiation			
Well and Moderate	1.00		
Poor	1.71	0.96 to 3.04	0.067
BCLC stage			
0 and A	1.00		
B	1.84	0.72 to 4.70	0.203
C	6.08	1.17 to 31.49	0.032*
Cirrhosis			
No	1.00		
Yes	0.48	0.25 to 0.92	0.027*
HBsAg			
Negative	1.00		
Positive	0.92	0.35 to 2.42	0.862
HBV-DNA (IU/ml)			
≤ 2000	1.00		
> 2000	0.73	0.33 to 1.60	0.424
Portal vein thrombosis			
No	1.00		
Yes	0.70	0.16 to 3.07	0.639
Tumor size (cm)			
< 5	1.00		
≥ 5	0.57	0.25 to 1.33	0.196
Number of tumors			
Single	1.00		
Multiple	0.94	0.44 to 2.04	0.883
Family history			
No	1.00		
Yes	1.07	0.45 to 2.58	0.873

Odds ratios and 95% CIs were calculated using multivariate logistic regression * indicates statistical significant ($P < 0.05$).
Abbreviations: CI, confidence interval.

Supplementary Table 4: Multivariate cox regression analysis for risk factors associated with disease-free survival of HCCs (*n* = 275)

Variables	Hazard ratio	95.0% CI	*P*
TERT promoter mutations			
Wild-type	1.00		
Mutant	1.72	1.23 to 2.39	0.001*
α-Fetoprotein (μg/L)			
≤ 200	1.00		
> 200	2.06	1.46 to 2.92	< 0.001*
Tumor differentiation			
Well and Moderate	1.00		
Poor	0.93	0.66 to 1.31	0.673
BCLC stage			
0 and A	1.00		
B and C	1.57	0.88 to 2.79	0.126
Portal vein thrombosis			
No	1.00		
Yes	2.18	1.40 to 3.39	0.001*
Tumor size (cm)			
< 5	1.00		
≥ 5	1.25	0.76 to 2.05	0.379
Number of tumors			
Single	1.00	1.14 to 2.47	
Multiple	1.68		0.008*

Hazard ratios and 95% CIs were calculated using multivariate Cox regression model.
*indicates statistical significant ($P < 0.05$). *Abbreviations*: CI, confidence interval.

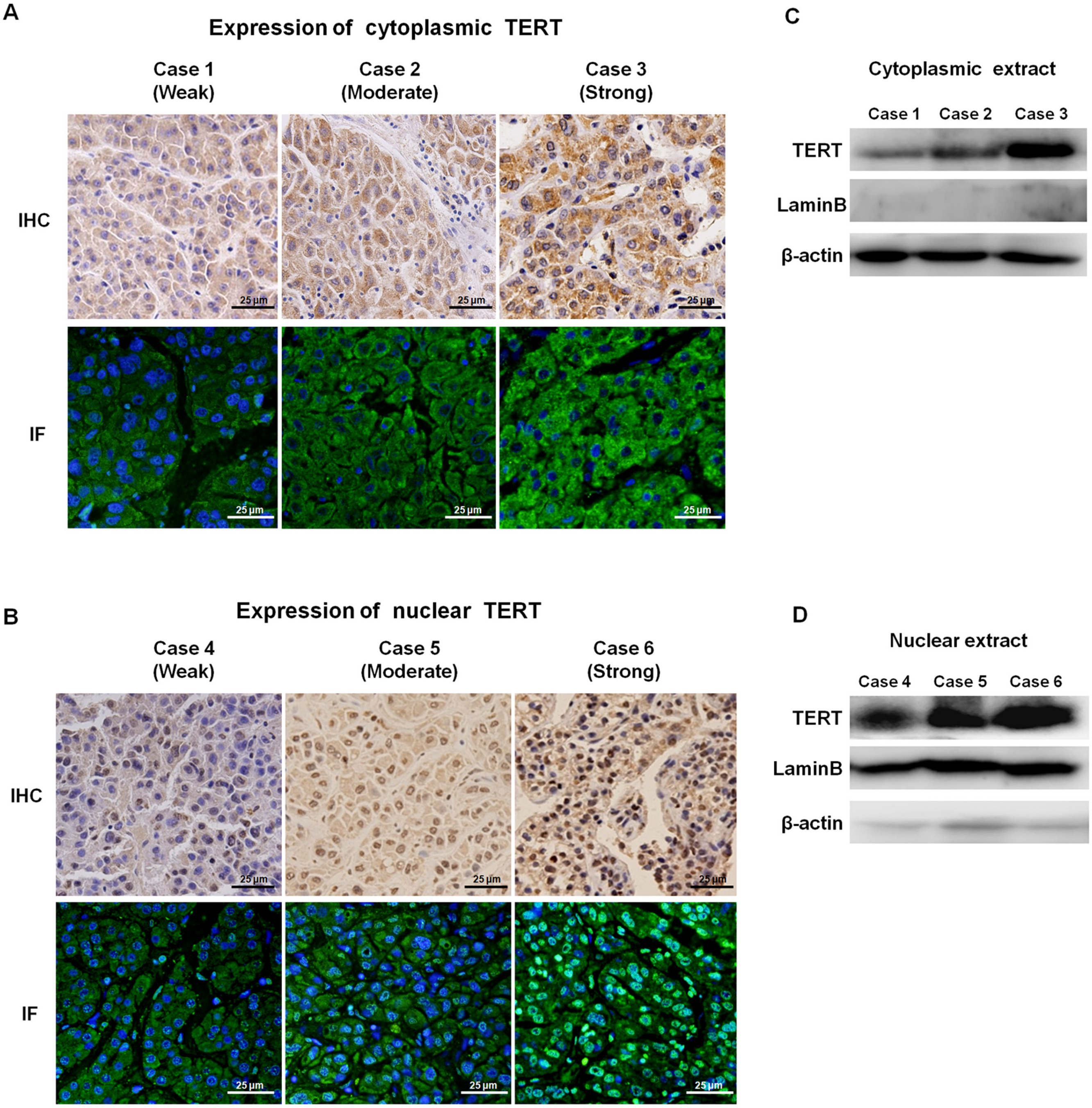

Supplementary Figure 1: TERT nuclear and cycoplasmic expression evaluated by immunohistochemistry, immunofluorescence and western blot. Representative images of IHC (immunohistochemical staining, upper panel) and IF (immunofluorescence staining, lower panel) of three cases of HCC demonstrated the expression level of TERT (weak, moderate and strong) in cytoplasm **(A)** and in nucleus **(B)**. TERT was probed with green fluorescence and the nucleus was stained with DAPI (blue). The cytoplasmic extract protein samples from case 1–3 and nuclear extract protein samples from case 4–6 were analyzed by western blot to confirm the expression level of cytoplasmic TERT **(C)** and nuclear TERT **(D)**. β-actin was used as cytoplasmic loading control for cytoplasmic extracts and laminB was used as nuclear loading control for nuclear extracts.
